# Lung Cancer Death Attributable to Long-Term Ambient Particulate Matter (PM_2.5_) Exposure in East Asian Countries During 1990–2019

**DOI:** 10.3389/fmed.2021.742076

**Published:** 2021-10-15

**Authors:** Xiaoxue Liu, Sumaira Mubarik, Fang Wang, Yong Yu, Yafeng Wang, Fang Shi, Haoyu Wen, Chuanhua Yu

**Affiliations:** ^1^Department of Epidemiology and Biostatistics, School of Public Health, Wuhan University, Wuhan, China; ^2^School of Public Health and Management, University of Medicine, Shiyan, China; ^3^Global Health Institute, Wuhan University, Wuhan, China

**Keywords:** death, lung cancer, YLL, PM_2.5_, elder, population attributable fraction (PAF)

## Abstract

**Background:** Ambient particulate matter is a public health concern in East Asia as it contributes to a growing number of all-cause and cancer deaths. This study aimed to estimate lung cancer death attributable to ambient particulate matter (PM) < 2.5 μm (PM_2.5_) in East Asia countries.

**Methods:** The attributable death rates of lung cancer were estimated based on the calculation of population attributable fraction. We performed joinpoint regression analysis and age-period-cohort (APC) model to estimate temporal trends of the attributable death to PM_2.5_.

**Results:** In 2019, PM_2.5_ was estimated to have caused 42.2% (nearly 0.13 million) of lung cancer deaths in East Asia men. During 1990–2019, the increase in age-standardized death rates of lung cancer attributable to PM_2.5_ was highest in China, which increased by 3.50% in males and 3.71% in females. The death rate caused by PM_2.5_ also significantly increased in the Democratic People's Republic of Korea (2.16% in males; 3.06% in females). Joinpoint analysis showed that the rates generally increased in younger and older people in both the Democratic People's Republic of Korea and Mongolia, while it only increased in elderly people in other countries'. Age effect from APC analysis demonstrated the risk of lung cancer death attributable to PM_2.5_ generally increased from young to old age. Period effect indicated that from 1994–1998 to 2019–2023 period risk continuously increased by 1.77, 1.68, and 1.72 times in China, the Democratic People's Republic of Korea, and Japan, respectively. The period risk decreased from 1999 to 2009 and subsequently increased from 2009 to 2019 in both the Republic of Korea and Mongolia.

**Conclusions:** The death rate of lung cancer attributable to PM_2.5_ is increasing in the Democratic People's Republic of Korea, Mongolia, and China. In East Asia, China is facing the highest attributable death rate in recent decades. The period effect suggested a remarkably increased risk of lung cancer death caused by PM_2.5_ in China, the Democratic People's Republic of Korea, and Japan during the long-term period. It is recommended that the governments of these countries should continuously concentrate on particulate matter pollution governance and improvement.

## Introduction

Ambient particulate matter of <2.5 μm (PM_2.5_) pollution is recognized as a major health concern worldwide. PM_2.5_ was associated with an increased risk of disease mortality and morbidities (1–4), and a 10-μg/m^3^ increment in PM_2.5_ was associated with a 4.3% increment in total mortality (5). However, ambient particulate matter pollution continues to be one of the largest increases in risk exposures from 1990 to 2019 worldwide (6).

In 2019, according to the Global Burden of Disease (GBD) 2019 study, among the 87 risk factors identified, long-term exposure to PM_2.5_ caused 118.2 million disability-adjusted life-years (DALYs), representing 4.7% of global DALYs in 204 countries and territories (7). Approximately 4,140,971 all-causes deaths and 307,680 lung cancer deaths in 2019 were attributed to ambient PM_2.5_ globally. However, the ambient PM_2.5_ risk-oriented health problems have mainly occurred in low- and middle-income countries (LMICs) according to previous studies (1, 8). In 2017, ambient particulate matter pollution ranked as the eighth leading risk for death, with a total of 2.94 million deaths globally and 1.05 million deaths in Southeast Asia, East Asia, and Oceania (2). Based on a newly published global comparative risk assessment in 2019, nearly all locations in south Asia, many parts of Southeast Asia, and most provinces in China had 10–15% of DALYs attributable to air pollution (6).

Lung cancer has been the leading cause of cancer deaths for many years worldwide, with the incidence and mortality markedly varying between countries (9, 10). Asia has extremely diverse lung cancer incidences, and it is the leading cause of cancer death in China (10, 11). The major risk factor for the burden of lung cancer was tobacco smoking, followed by air pollution-related age-standardized rates (12). This study aims to focus on lung cancer death caused by particulate matter pollution. Previous studies have evaluated the association of ambient air pollution with lung cancer (13–15). Few studies have been conducted regarding ambient air pollutant-related lung cancer death (16), while no national study has comprehensively evaluated the long-term trend in lung cancer death due to long-term exposure to ambient particulate matter in East Asia countries. Although a recent study reported that the age-standardized death rate attributable to air pollution decreased by 60.6% for China overall between 1990 and 2017 (17), the temporal trend in lung cancer death attributable to air pollution was not clearly analyzed. Therefore, this study aimed to estimate the trend and risk for long-term exposure to PM_2.5_ caused by lung cancer death from 1990 to 2019 among East Asia countries, including China, Mongolia, the Democratic People's Republic of Korea, the Republic of Korea, and Japan.

## Materials and Methods

### Data Sources and Methods for Quantifying the Health Burden

The attributable burden of lung cancer data (1990–2019) for China, the Democratic People's Republic of Korea, the Republic of Korea, Japan, and Mongolia was collected from the most recent GBD project. The datasets analyzed during the current study are published and available in the Global Burden of Disease Database repository. Due to the publicly available nature of data, ethical approval and patient consent to participate were not required in this study.

The GBD 2019 provides a systematic scientific assessment of published, publicly available, and contributed data on incidence, prevalence, mortality, and risk factors for a mutually exclusive and collectively exhaustive list of diseases and injuries. It contains data on 87 behavioral, environmental, occupational, and metabolic risks or clusters of risks, 369 diseases and injuries, and the healthy life expectancy (HALE) for 204 countries and territories (http://ghdx.healthdata.org/).

The original data on lung cancer death were obtained from the local Center for Disease Control and Prevention (CDC), Disease Surveillance Points (DSPs), and the Maternal and Child Surveillance System. Ambient air quality was estimated by extracting the annual average mass concentration data of ambient PM_2.5_ from the multiple-source data, including satellite observation, ground measurement, and chemical migration model simulation, etc., and the grid-level exposure to ambient PM_2.5_ was estimated by data integration model for air quality (DIMAQ) (6, 18). The relative risks (RR) of ambient particulate matter pollution at different exposure levels for different health outcomes were estimated as the Integrated Exposure Response function of exposure based on 81 published systematic reviews, the specific methods are outlined in a previous study (6).

Population attributable fraction (PAF) was defined as if the exposure of a certain risk factor was reduced to the theoretical minimum exposure level in a certain population, the proportion of related diseases or deaths in the population would reduce (2, 19). In this study, the exposure level associated with minimum risk, known as the theoretical minimum risk exposure level, for ambient particulate matter pollution was between 2.4 and 5.9 μg/m^3^. Lung cancer deaths caused by PM_2.5_ were estimated based on defining PAF through combining the distribution of exposure to air pollution with exposure-risk estimates at each level of exposure.


(1) $$\begin{array}{l} PAF=\frac{\sum_{i}^{n}p_{i}\left(RR_{i}-1\right)}{\sum_{i}^{n}p_{i}\left(RR_{i}-1\right)+1} \end{array}$$

where *P*_*i*_ is the percentage of the population exposed to level *i* of ambient air exposure, *RR*_*i*_ is the relative risk at exposure level *i*, and *n* is the total number of exposure levels.

Attributable death numbers were computed by multiplying PAFs by the relevant outcome quantity for each age-sex-location-year (6, 20). For example, the attributable deaths (AD) were calculated by multiplying the number of the deaths for lung cancer (N) and the PAF:


(2) $$\begin{array}{l} AD=PAF\times N \end{array}$$

The age-standardized rate of attributable deaths caused by PM_2.5_ was calculated by the world standard population (21).

### Statistical Analysis

#### Joinpoint Regression Analysis

Temporal trends for lung cancer death rates attributable to PM_2.5_ were assessed using the joinpoint regression analysis (Joinpoint regression software, Version 4.5.0.1, available through the Surveillance Research Program of the United States National Cancer Institute). Joinpoint analysis was based on regression with age-standardized mortality rates as the dependent variables and with year as the independent variable. Gender and age group were the by-variables. In the model, logarithmic transformation of the rates was carried out and the standard errors were calculated based on binomial approximation (22). An average annual percentage of change (AAPC), along with the corresponding 95% confidence interval (95%CI), was calculated for each trend. The statistically significant AAPC values indicated a temporal change in mortality trend over the time period. We used the Monte Carlo Permutation method for tests of significance. In this analysis, age groups under 25 years old were excluded due to very low probabilities.

#### Age-Period-Cohort Analysis

APC analysis could decompose the accumulation of health risks for incidence and death of disease (23). This analysis is developed to reflect the relative risks of disease by estimates on age, period, and cohort effects. Age effect indicates that the risk of death from disease increases with the process of aging. The period effect represents influential factors that simultaneously affect all age groups with advanced periods. The cohort effect represents variations across groups of individuals born in the same year or years. These effects influence morbidity and mortality risk of diseases in specific ways. An intrinsic estimator (IE) method used in this APC analysis could decompose the three effects simultaneously. In this model, the death rates of lung cancer attributed PM_2.5_ were recoded into successive 5-year age groups (25–29, 30–34, …, 90–94), consecutive 5-year periods (1994–1998, 1999–2003, 2004–2008, 2009–2013, 2014–2018, 2019–2023) and correspondingly consecutive 5-year birth cohort groups (1904–1908, 1909–1913, …, 1994–1998).

The estimated coefficients of age, period, and cohort effects are plotted in **Figure 2**, and their exponential values [exp(coef.) = e^coef.^] denote the relative risk (RR) of the age, period, or birth cohort effect. Age, period, and cohort effects were analyzed by Stata 12.0 software (StataCorp, College Station, TX, USA). Deviance, Akaike Information Criterion (AIC), and Bayesian Information Criterion (BIC), and represent the loss of information caused by using models to represent the process of generating the actual data.

## Results

### Lung Cancer Attributable Death to PM_2.5_ in East Asia Countries for Males and Females

In 2019, there were 2,042,639 deaths and 2,259,998 new cases of lung cancer worldwide, with a 25.18/100,000 ASDR and 27.66/100,000 ASIR. Importantly, a significant number of these 1,508,993 deaths were attributed to ambient particulate matter pollution that occurred in these East Asia countries, accounting for 36.4% of the global deaths attributed to ambient particulate matter pollution, and 12.3% of the attributed deaths were due to lung cancer. The death rate of lung cancer attributable to PM_2.5_ among men was 2.34 times higher than that in women.

The global number of lung cancer deaths due to ambient particulate matter pollution were 307,680, 60.1% (185,041 deaths) of which were concentrated in East Asia countries, especially in male patients (42.2%). Table 1 shows lung cancer attributable death to PM_2.5_ in East Asia countries for both male and female patients.

**Table 1:** Lung cancer death and YLL attributable to PM_2.5_ in East Asia in 1990 and 2019.

**East Asia**	**1990**				**2019**			
	**Death**		YLL^*^		**Death**		YLL^*^	
	**Number**	**Age-standardized**	**Number**	**Age-standardized**	**Number**	**Age-standardized**	**Number**	**Age-standardized**
		**Per 100,000**		**Per 100,000**		**Per 100,000**		**Per 100,000**
**Male**								
China	20,576.26	5.42	548,863.25	123.69	120,186.42	13.36	2,704,360.07	271.65
Democratic People's Republic of Korea	243.67	4.09	7,097.63	99.15	899.90	7.04	23,694.74	164.94
Republic of Korea	862.99	7.06	24,070.02	167.94	3,085.32	8.31	60,056.13	147.69
Japan	2,709.53	3.89	57,200.87	76.31	5,513.54	3.21	88,070.16	58.29
Mongolia	28.30	6.55	695.62	144.09	95.61	11.43	2,518.83	240.23
**Female**								
China	7,060.43	1.69	183,062.82	40.27	51,113.34	4.99	1,107,912.93	105.28
Democratic People's Republic of Korea	85.01	0.89	2,288.95	22.18	367.73	1.94	8,661.43	47.23
Republic of Korea	300.29	1.82	7,878.83	42.89	1,240.90	2.43	21,475.66	44.36
Japan	1,054.48	1.09	21,248.93	22.47	2,516.99	1.02	34,851.02	19.27
Mongolia	6.40	1.17	149.19	26.21	24.35	2.28	577.58	45.55

* *YLL, Years of Life Lost*.

**Figure 1:**
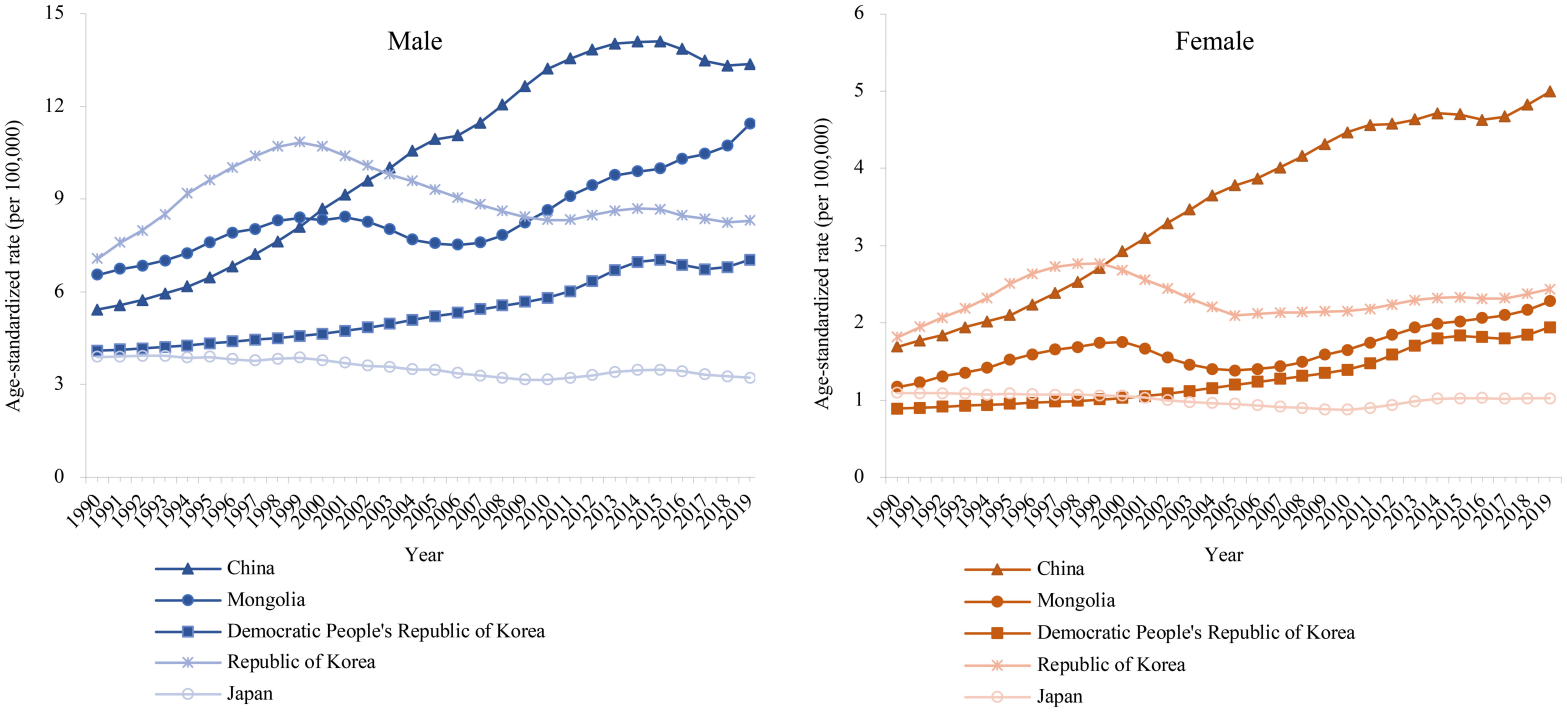
The age-standardized rates of lung cancer attributable death to PM_2.5_ in East Asia for both sexes at all ages, during 1990–2019.

Figure 1 shows the secular trends of the age-standardized rate of lung cancer death attributable to PM_2.5_ in East Asia during 1990–2019. We observed a significant increase in the death rate among patients in China, the Democratic People's Republic of Korea, and Mongolia. We found a continuously decreasing trend in Japan for both male and female patients. In contrast, the death rate from the Republic of Korea peaked in 1999 while a slight rise in female patients has been observed since 2005.

#### Results of Joinpoint Regression Analysis

We conducted Joinpoint regression analysis to estimate the average annual percent changes in age-standardized and age-specific death rates of lung cancer attributable to PM_2.5_ in East Asia (Table 2). Between 1990 and 2019, the increase in the death rate to PM_2.5_ was highest in China [AAPC: 3.50 [95% CI, 3.05–3.94] in male and 3.71 [95% CI, 3.26–4.17] in female patients], and a small decrease was observed in Japan (AAPC: −0.76 [95% CI, −0.92 to −0.60] in male and −0.43 [−0.67 to −0.18] in female patients). As a high SDI country, the Republic of Korea showed no obvious change in the death rate. Similar to China, there was also a substantial increase in the Democratic People's Republic of Korea over the past 30 years (AAPC: 2.16 [95% CI, 2.01–2.32] in male and 3.06 [2.83–3.30] in female patients). We observed a slight increase in Mongolia, and the average percent changes increased by 1.63 (1.19–2.07) annually.

**Table 2:** The average annual percent changes (AAPC) in lung cancer deaths attributable to PM_2.5_ in East Asia during 1990–2019 for male and female patients.

ASR^*^ and Age-group (year)	**Average annual percent changes (AAPC), male**					**Average annual percent changes (AAPC), female**				
	**China** **(95% CI)**	**Democratic people's republic of korea** **(95% CI)**	**Republic of korea** **(95% CI)**	**Japan** **(95% CI)**	**Mongolia** **(95% CI)**	**China** **(95% CI)**	**Democratic people's republic of korea** **(95% CI)**	**Republic of korea** **(95% CI)**	**Japan** **(95% CI)**	**Mongolia** **(95% CI)**
ASR	3.50 (3.05, 3.94)	2.16 (2.01, 2.32)	−0.35 (−0.81, 0.12)	−0.76 (−0.92, −0.60)	1.52 (1.23, 1.81)	3.71 (3.26, 4.17)	3.06 (2.83, 3.30)	−0.07 (−0.52, 0.39)	−0.43 (−0.67, −0.18)	1.63 (1.19, 2.07)
25–29	−1.28 (−1.52, −1.04)	1.63 (1.35, 1.92)	−4.46 (−4.99, −3.94)	−2.07 (−2.26, −1.87)	1.09 (0.56, 1.62)	−2.83 (−3.77, −1.87)	2.84 (2.57, 3.11)	−3.55 (−4.02, −3.08)	−1.64 (−1.87, −1.40)	2.92 (2.68, 3.16)
30–34	−1.25 (−1.49, −1.01)	1.24 (0.95, 1.53)	−3.65 (−4.07, −3.23)	−2.81 (−3.05, −2.57)	2.93 (2.62, 3.23)	−2.73 (−3.43, −2.02)	2.66 (2.42, 2.90)	−2.86 (−3.18, −2.53)	−2.09 (−2.37, −1.80)	0.79 (0.31, 1.28)
35–39	−1.88 (−2.18, −1.58)	0.87 (0.68, 1.06)	−3.47 (−3.83, −3.11)	−2.78 (−2.98, −2.58)	1.97 (1.63, 2.30)	−2.01 (−2.33, −1.70)	2.55 (2.34, 2.76)	−1.84 (−2.04, −1.63)	−2.22 (−2.43, −2.00)	1.11 (0.57, 1.65)
40–44	−1.81 (−2.08, −1.54)	1.01 (0.85, 1.18)	−3.50 (−3.69, −3.30)	−2.66 (−2.93, −2.38)	1.54 (1.21, 1.87)	−1.87 (−2.13, −1.61)	2.52 (2.29, 2.76)	−1.89 (−2.12, −1.66)	−2.27 (−2.59, −1.95)	0.62 (1.12, 2.56)
45–49	−1.07 (−1.33, −0.81)	1.39 (1.22, 1.57)	−3.66 (−3.89, −3.44)	−2.29 (−2.58, −1.99)	1.78 (1.45, 2.11)	−2.01 (−2.47, −1.54)	2.71 (2.49, 2.94)	−1.25 (−1.35, −1.15)	−2.06 (−2.37, −1.74)	1.32 (0.80, 1.85)
50–54	−1.15 (−1.42, −0.88)	1.61 (1.44, 1.77)	−3.56 (−3.75, −3.38)	−1.31 (−1.60, −1.01)	2.00 (1.85, 2.16)	−1.44 (−1.72, −1.15)	2.86 (2.62, 3.10)	−1.32 (−1.60, −1.04)	−1.53 (−1.82, −1.23)	−0.12 (−0.74, 0.51)
55–59	−0.61 (−0.78, −0.43)	1.96 (1.82, 2.10)	−3.11 (−3.39, −2.82)	−0.92 (−1.07, −0.77)	1.63 (1.33, 1.94)	−0.78 (−1.12, −0.44)	2.99 (2.74, 3.24)	−1.44 (−1.65, −1.24)	−0.93 (−1.07, −0.79)	−0.31 (−0.96, 0.35)
60 - 64	0.05 (−0.14, 0.24)	2.03 (1.89, 2.18)	−2.42 (−2.71, −2.12)	−0.84 (−1.13, −0.56)	1.61 (1.16, 2.05)	−0.45 (−0.57, −0.34)	2.96 (2.70, 3.22)	−1.36 (−1.51, −1.20)	−0.37 (−0.55, −0.19)	0.10 (−0.49, 0.69)
65–69	0.26 (0.07, 0.45)	2.29 (2.13, 2.44)	−1.59 (−1.97, −1.21)	−1.05 (−1.39, −0.70)	0.45 (−0.09, 0.99)	−0.25 (−0.44, −0.08)	2.99 (2.73, 3.25)	−1.35 (−1.69, −1.00)	−0.28 (−0.58, 0.02)	0.63 (0.06, 1.21)
70–74	0.57 (0.32, 0.83)	2.44 (2.25, 2.62)	−0.53 (−1.08, 0.02)	−1.29 (−1.61, −0.97)	0.69 (0.19, 1.19)	0.30 (−0.04, 0.65)	3.07 (2.83, 3.31)	−0.82 (−1.36, −0.28)	−0.64 (−0.98, −0.29)	2.03 (1.56, 2.51)
75–79	1.13 (0.81, 1.46)	2.51 (2.35, 2.67)	0.46 (−0.29, 1.22)	−1.32 (−1.47, −1.17)	0.79 (0.35, 1.23)	0.99 (0.64, 1.33)	3.22 (2.98, 3.46)	−0.18 (−0.89, 0.54)	−0.97 (−1.30, −0.65)	1.64 (1.25, 2.04)
80–84	1.77 (1.39, 2.15)	2.46 (2.29, 2.62)	1.62 (0.74, 2.52)	−0.44 (−0.58, −0.29)	2.20 (1.83, 2.58)	1.78 (1.43, 2.13)	3.37 (3.16, 3.57)	0.80 (−0.17, 1.78)	−0.38 (−0.68, −0.07)	2.86 (2.55, 3.17)
85–89	2.57 (2.15, 2.98)	2.37 (2.22, 2.53)	2.88 (2.19, 3.57)	0.46 (0.28, 0.64)	3.87 (3.67, 4.07)	1.65 (1.36, 1.95)	3.59 (3.42, 3.77)	2.50 (1.93, 3.09)	0.31 (0.08, 0.54)	4.28 (4.01, 4.56)
90–94	2.08 (1.66, 2.50)	2.17 (2.06, 2.29)	1.97 (1.36, 2.59)	1.15 (1.04, 1.25)	4.19 (3.89, 4.49)	1.37 (1.17, 1.56)	3.75 (3.56, 3.94)	2.72 (2.18, 3.26)	1.53 (1.38, 1.68)	5.52 (5.08, 5.96)

* *ASR, age-standardized rate, which was age-standardized by the GBD 2019 global age-standard population; CI, Confidence interval*.

We also estimated changes in the age-specific rates of lung cancer death attributed to PM_2.5_ across age groups (from 25–29 to 90–94 age group) (Table 2). We noted that the death rates in all age groups rose significantly in both the Democratic People's Republic of Korea and Mongolia. Other East Asia counties showed declines in the death rate for younger age groups but increases in older age groups. In China, the death rate to PM_2.5_ rose significantly among people aged 65 years and over for male patients, and among people aged 75 years and over for female patients. In Japan, for both sexes, the rate generally declined for most age groups except for 85–89 and 90–94 age groups. In the Republic of Korea, an increase in the rate was also observed in elderly age groups (≥75 years in males and ≥80 years in females). Therefore, lung cancer death due to PM_2.5_ generally increased in the Democratic People's Republic of Korea and Mongolia but decreased for all age groups in Japan. We almost observed a significant increase among elderly people aged 75 years and above in China and the Republic of Korea.

#### Results of Age-Period-Cohort Analysis

**Figure 2:**
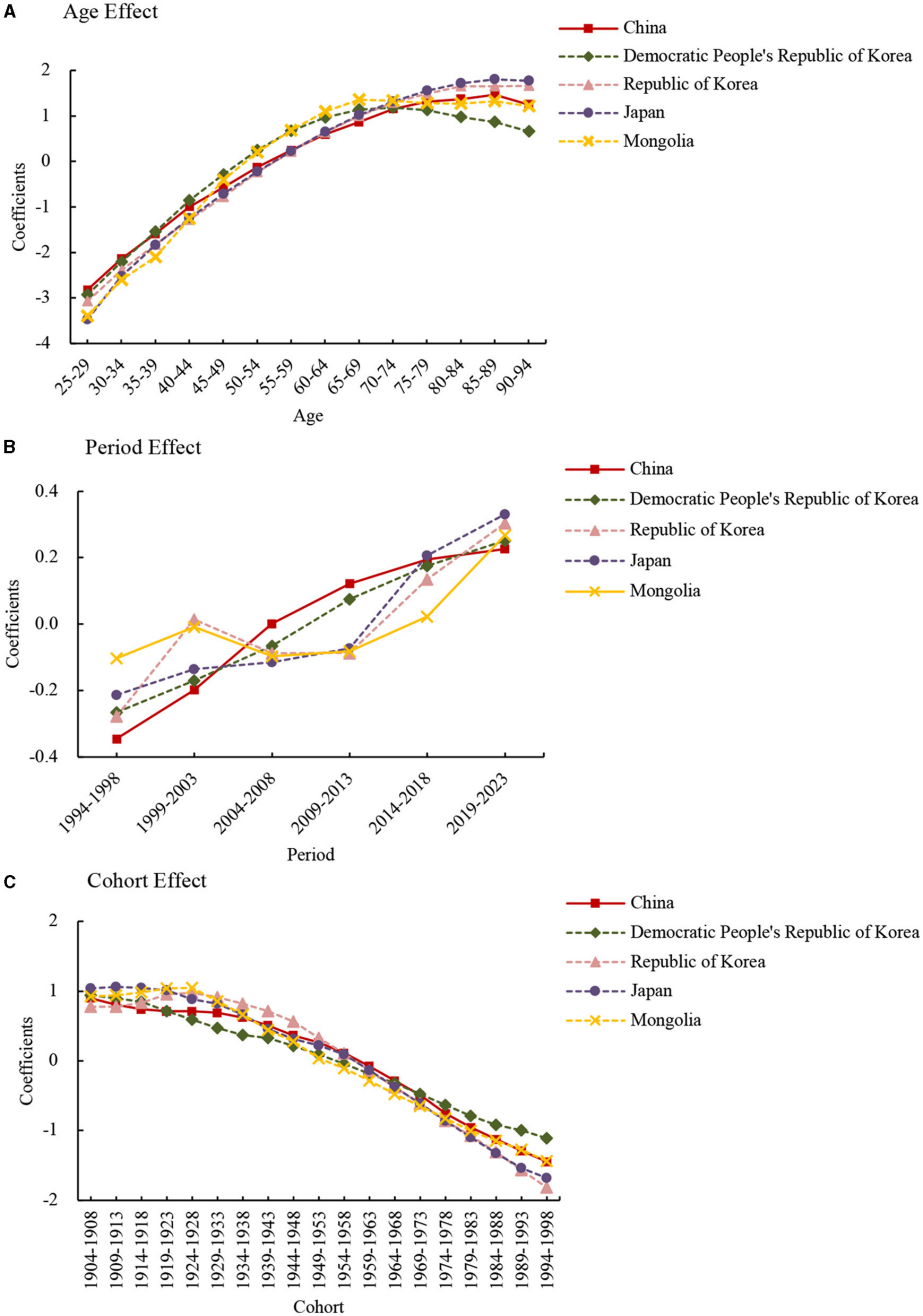
Lung cancer death attributable to PM_2.5_ estimated coefficients for the age **(A)**, period **(B)**, and cohort effects **(C)**.

The coefficients of age effect on lung cancer death attributed to PM_2.5_ increased from the 25–29 to 90–94 age group in East Asia countries, except for in the Democratic People's Republic of Korea, where the age risk peaked in the 70–74 age group (Figure 2A). The age effect showed that in the from 25–29 to 90–94 age group, the risk for lung cancer death attributed to PM_2.5_ increased by 58.99, 36.51, 113.04, 190.68, and 101.86 times in China, the Democratic People's Republic of Korea, Republic of Korea, Japan, and Mongolia, respectively.

The period effect showed different trends in the death rates among East Asia countries (Figure 2B). In the from 1994–1998 to 2019–2023 period, the risk increased by 1.77, 1.68, and 1.72 times in China, the Democratic People's Republic of Korea, and Japan, respectively. The period effect in the Republic of Korea and Mongolia showed an N-shape trend, which decreased from 1999 to 2009 and increased from 2009 to 2019.

The cohort effect showed an ongoing decreasing trend from 1904–1908 to 1994–1998 birth cohort in all these countries (Figure 2C). From the earliest birth cohort to the most recent cohorts, the risk decreased by around 90% for all East Asia counties.

### Lung Cancer Attributable YLL to PM_2.5_ in East Asia Countries

**Figure 3:**
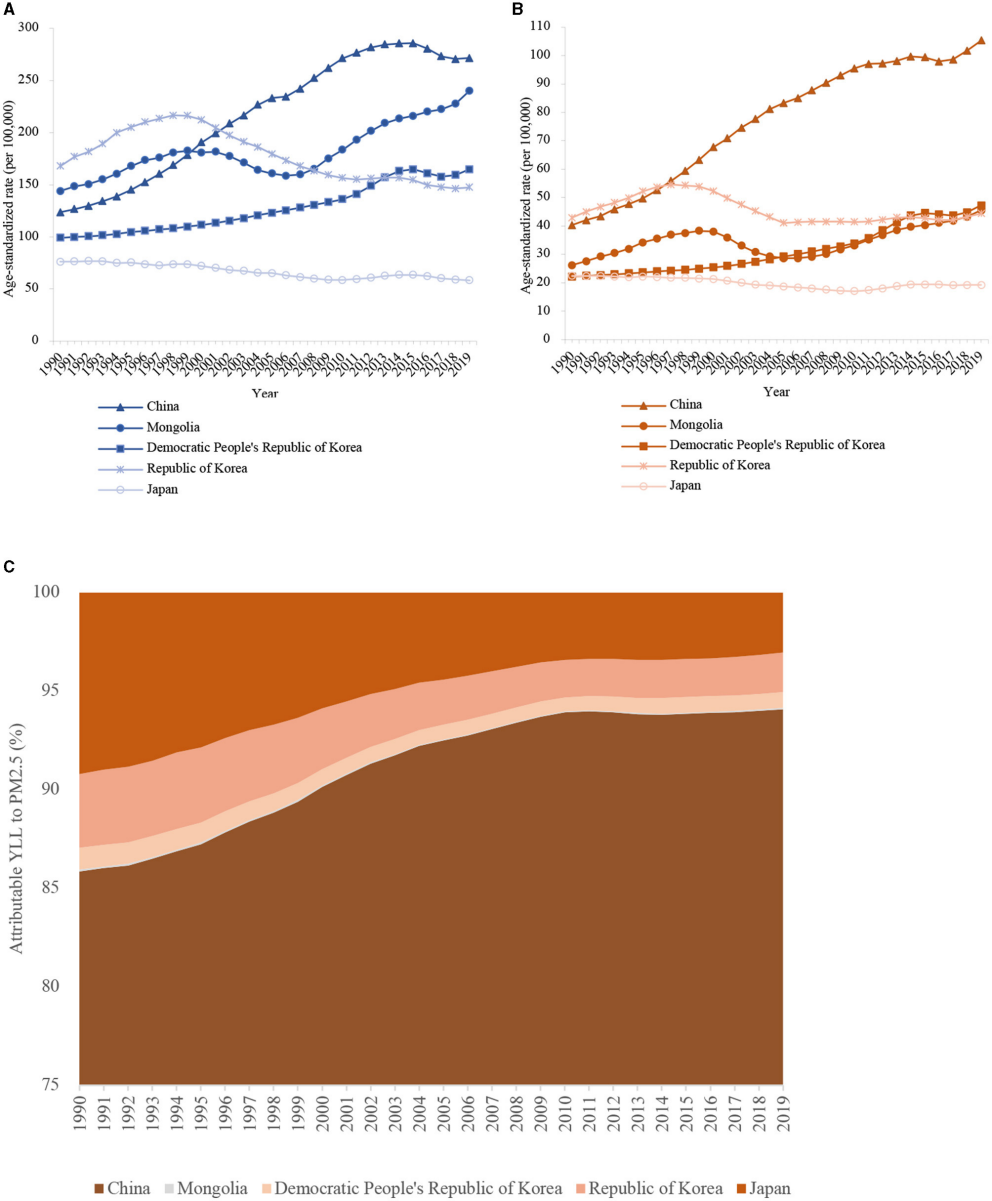
The age-standardized rates of lung cancer attributable YLL to PM_2.5_ in East Asia for both sexes at all ages, during 1990–2019. **(A)** male patients; **(B)** female patients; **(C)** The proportion of lung cancer YLL attributed to ambient PM_2.5_ in East Asia countries from 1990 to 2019.

The YLL rate of lung cancer attributable to PM_2.5_ also showed a significant increase for male and female patients in the three regions (Figure 3).

In 2019, in male patients, the age-standardized YLL rate of lung cancer attributed to PM_2.5_ was highest in China (271.65/100,000), closely followed by Mongolia (240.23/100,000) (Table 1). The YLL rate to PM_2.5_ was relatively lower in the Democratic People's Republic of Korea (164.94/100,000) and the Republic of Korea (147.69/100,000). The YLL rate to PM_2.5_ was lowest in Japan (58.29/100,000). In females, the highest YLL rate was observed in China (105.28/100,000) and the lowest rate in Japan (19.27/100,000). Overall, the YLL rates of lung cancer due to PM_2.5_ were much lower in females than males among all East Asia countries. Compared with 1990, the age-standardized YLL rate of lung cancer attributed to PM_2.5_ increased by 1.20 and 1.61 times in male and female patients in China, respectively (Table 1). However, the age-standardized YLL rate of lung cancer attributed to PM_2.5_ decreased in male (24.0%) and female (14.0%) patients in Japan. In the Republic of Korea, the rate decreased by 12.0% in male and increased by 3.0% in female patients.

We also plotted the distribution proportion of lung cancer YLL attributed to ambient PM_2.5_ in East Asia from 1990 to 2019 (Figure 3C). The YLLs from East Asia were mainly driven by China. Indeed, 94.08, 0.08, 0.80, 2.01, and 3.03% of YLLs attributable to PM_2.5_ in 2019 were due to lung cancer in China, Mongolia, the Democratic People's Republic of Korea, the Republic of Korea, and Japan, respectively. Overall, apart from death, China also has a substantially increased lung cancer YLL attributed to PM_2.5_ and mainly contributed to the YLLs from East Asia.

### Lung Cancer Attributable Death and YLL to PM_2.5_ in East Asia Compared With the Global Level in 2019

**Figure 4:**
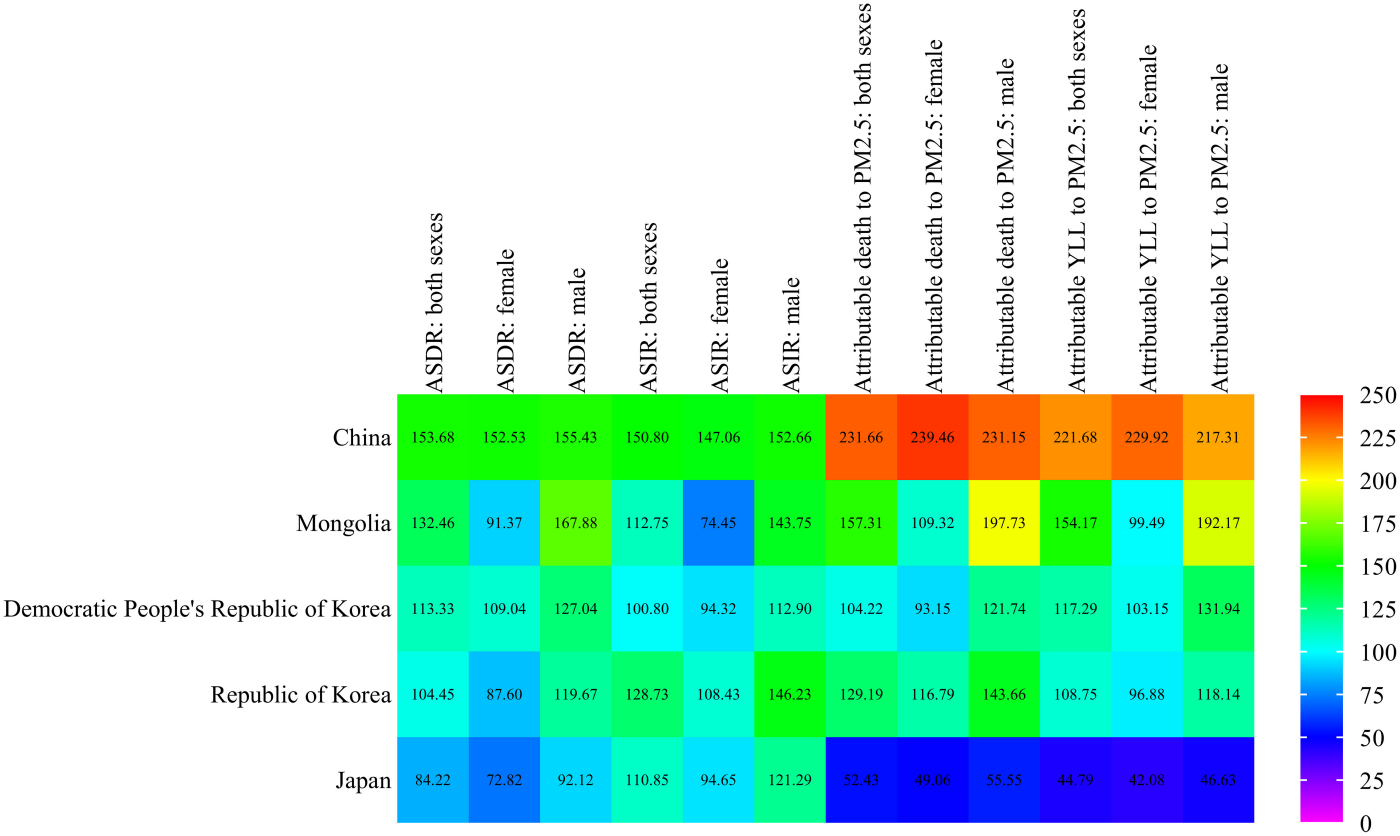
Age-standardized rates of lung cancer attributable death and YLL to PM_2.5_ in East Asia compared with those globally in 2019.

Compared with the global level, we plotted the ASDR, ASIR, and death and YLL attributable to the PM_2.5_ of lung cancer in East Asia countries (Figure 4 and Supplemental Table 1). The ASDR and ASIR of lung cancer in these East Asia countries were about 1.0–1.5 times higher than those of the global average in both sexes, while the low ASDR of Japan was 0.84 times that of the global average. In addition, the ASDR and ASIR of lung cancer were higher in male than female patients. In 2019, the death and YLL rates of lung cancer attributable to PM_2.5_ in China were more than 2.0 times higher than those of the world average. In Mongolia, the rates were around 1.5 times that of global levels. It was 1.0–1.3 times more than global levels in the Democratic People's Republic of Korea and the Republic of Korea than the global level, while the level differed in the two countries. There were about 0.4 and 0.5 times higher levels in Japan for lung cancer death and YLL attributable to PM_2.5_. In terms of genders, the death and YLL rates of lung cancer attributed to PM_2.5_ in male patients were higher than that in female patients for most East Asia countries when compared with global levels. The gender difference was exactly different for China (Attributable to death: 231.15% in male patients, and 239.46% in female patients; Attributable to YLL: 217.31% in male patients, 229.92% in female patients). Generally, compared with the global level, China had the highest attributable death and YLL of lung cancer due to PM_2.5_ in East Asia.

## Discussion

This study comprehensively estimates on temporal trends in lung cancer death caused by long-term exposure to ambient PM_2.5_ in East Asia. We found that the lung cancer attributable death caused by PM_2.5_ generally increased in younger and older people in both the Democratic People's Republic of Korea and Mongolia by Joinpoint analysis. APC analysis demonstrated that the risk of lung cancer death attributed to PM_2.5_ generally increased from young to old age, and the risk increased continuously in China, the Democratic People's Republic of Korea, and Japan from 1994–1998 to 2019–2023 period. The Republic of Korea and Mongolia showed a different period risk trend. The cohort effect declined to the most recent birth cohorts, which demonstrated a decreasing risk from the old generation to the new generation.

This section discusses the attributable death of lung cancer due to ambient particulate matter. Among East Asia countries, we found that China and Mongolia had high mortality and YLL rates attributable to ambient PM_2.5_. The high population density in China exhibited a huge lung cancer burden attributed to ambient PM_2.5_ and was a major contributor to the death and YLL burden caused by PM_2.5_ in East Asia. This finding was related to the fact that more than a quarter of global deaths caused by ambient particulate matter pollution occurred in China, which is the third risk factor affecting the health of Chinese people (16). A huge proportion of attributable YLL to PM_2.5_ was related to China, which is consistent with studies by the World Bank and the WHO. The Chinese Academy for Environmental Planning on the effect of air pollution on health concluded that between 350,000 and 500,000 people die prematurely each year as a result of outdoor air pollution in China (18, 24). This finding could be contributed to the fact that PM_2.5_ pollution in the winter is worse in China (4), in addition to the increasing high mortality of lung cancer in China (7). Mongolia has a relatively high level of age-standardized attributable death and YLL rates. This is because, during the cold wintertime in Mongolia, a lot of coal is burnt for domestic heating. As reported, ambient particulate levels frequently exceed 100 times the WHO-recommended safety level for sustained periods and account for the majority of personal particulate matter exposure (25). Reducing home heating emissions in traditional housing areas has been the primary focus of air pollution control efforts for Mongolian cities (26). Moreover, we observed that Mongolia still has a much higher level of lung cancer mortality in 2019, which is only second to China, although it has been decreasing over the past decades. Differing from China and Mongolia, Japan's age-standardized attributable death and YLL rates were the lowest from 1990 to 2019. This may be related to the fact that Japan has a declining relatively low level of air pollution, and its mortality rate from lung cancer is relatively low compared with other Asian countries (14, 27). A relatively low level of attributable burden was observed in both the Democratic People's Republic of Korea and the Republic of Korea, while the difference was that the death and YLL rate of lung cancer due to PM_2.5_ increased in the former but declined in the latter. Ambient air pollution is recognized as a major environmental health hazard in the Republic of Korea and the average PM_2.5_ exposure concentration for the whole period was estimated to be 30.2 μg/m^3^ during 1990–2013 (28). However, the mortality rate from lung cancer was much lower than in other countries. Therefore, although the Republic of Korea's air pollution is relatively high, the lung cancer attributable burden related to PM_2.5_ is not at a high level. During 1990–2019, lung cancer death and YLL rates to PM_2.5_ have increased in China, the Democratic People's Republic of Korea, and Mongolia, and decreased in Japan and the Republic of Korea. In East Asia countries, these trends were consistent with mean PM_2.5_ concentrations, which showed substantial increases in China, with decreases observed in Japan during 1990–2013 (29).

We observed gender and area-specific distributions of lung cancer attributable burdens due to PM_2.5_. Lung cancer deaths caused by ambient particulate matter were 2.34 times higher in East Asia men than women in 2019. Furthermore, the age-standardized death and YLL rates of lung cancer attributed to PM_2.5_ were much higher in men than women in all countries, which was consistent with the gender difference in trends of lung cancer mortality. A previous study also reported that long-term PM_2.5_ exposure has led to a much higher number of lung cancer premature death in male patients than in female patients (28).

In East Asia, the YLL burden of lung cancer caused by PM_2.5_ was also mainly driven by China, especially in men. This finding was consistent with another previous study, which indicated that the elderly and men had higher health risks than younger people and women. When the PM_2.5_ concentrations meet the WHO air quality guidelines of 10 μg/m3, 84% of the premature deaths would be avoided (30). Compared to 1990, the age-standardized death rate of lung cancer attributed to PM_2.5_ increased in 2019, while the increase rate was smaller in males than females in the Democratic People's Republic of Korea, Republic of Korea, and Mongolia. Outdoor air pollution occurs predominantly in developing countries, particularly in Asia (1, 31). In China, industrial and residential sources were the two leading sources of mortality due to exposure to ambient PM_2.5_ (32), and annual concentrations of ambient PM_2.5_ are more than 5 times higher than the WHO guideline values in many populous cities. In Mongolia, sources concentrated on increased coal consumption in the cold season (33). Whether females were exposed to a higher risk of death in these countries, need to be further explored in relation to the different sources of mortality caused by ambient PM_2.5_. In Japan, we observed that lung cancer death attributable to PM_2.5_ decreased in both sexes. Increasing automobile traffic has caused considerable increases in concentrations of particulate matter, and concentrations have gradually decreased since control measures based on the automobile PM law were enforced in 2001 (34).

It is well-known that the major risk factor for lung cancer is tobacco smoking (12). Reactive oxygen or nitrogen species (ROS, RNS) and oxidative stress in the respiratory system could initiate or promote mechanisms of carcinogenesis, and the lungs are likely exposed to oxidants generated exogenously (air pollutants, cigarette smoke, etc.) daily (35). As reported, inhalable quartz, metal powders, mineral asbestos fibers, ozone, soot from gasoline and diesel engines, tobacco smoke, and ambient PM are involved in various oxidative stress mechanisms (35–38). It seems to be highly possible that PM_2.5_ not only adds to lung cancer risk but also smoking habits over the years might be related to some of the differences of lung cancer, especially for men and women. Because smoking significantly increases the risk of lung cancer, which has been reported to be the main cause of lung cancers in 90% of male and 79% of female patients (39, 40). China is still facing heavy tobacco use, and lung cancer is the leading cause of cancer death and morbidity. A series of policies to control tobacco consumption and prevent lung cancer have been carried out. However, the burden of lung cancer is still serious, and the smoking rate in men is still very high (41).

In terms of age difference, the attributable death to PM_2.5_ of lung cancer generally increased among elderly people (age ≥75 years) in both China and the Republic of Korea. This finding was also observed in other studies, which showed that long-term exposure to PM_2.5_ was associated with an increased risk of disease death among people aged 65 years and older (42, 43). However, it is noteworthy that lung cancer death attributable to PM_2.5_ increased for all age groups in the Democratic People's Republic of Korea and Mongolia. We also found that the percentage change of ASR of lung cancer death caused by PM_2.5_ for China was significantly higher than that in any age groups in Table 2. This result was related to the age-standardized rate, which eliminates the influence of different age components of the population, and ensures the comparability of the lung cancer death rate.

Days of heavy pollution regularly occur in Asian megacities (23). China and the Democratic People's Republic of Korea had increasing attributable deaths rates of lung cancer caused by PM_2.5_, and the period risk has also increased over the past few decades. According to the most up-to-date risk factor assessment, global exposure to harmful environmental risks has been declining, with the notable exception of ambient particulate matter pollution (6). In China, a large proportional increase in PM_2.5_ was observed between 1990 and 2013 (29). While the early air pollution policies were ineffective at reducing emissions since the 1980s, and air pollution problems dominated by PM_2.5_ have emerged and worsened since 2005 (44). As PM_2.5_ was not been included in the National Air Reporting System until 2013, we collected data on the national annual mean PM_2.5_ concentrations in China and found that it declined with a range of 72–36 μg/m^3^ from 2013 to 2019 (Supplemental Figure 1). Another previous study also reported that the average annual population-weighted PM_2.5_ exposure in China was 52.7 μg/m^3^ in 2017, which is 9% lower than in 1990 (17). This decline is because of effective air pollution control policies after the winter-long PM_2.5_ episode in China (44). However, the annual average PM_2.5_ concentration in China was 36 μg/m^3^ in 2019 and the planned reductions in annual average PM_2.5_ concentration from the current level to 10 μg/m^3^ still have not been achieved. As early as 2013, 87% of the global population lived in areas exceeding the WHO air quality guideline of 10 μg/m^3^ PM_2.5_ (annual average) (29). Thus, the changes derived from the policy evolution have implications for future studies, as well as further reforming the management of health risk and air quality control. More interventions are required to achieve the guideline levels of PM_2.5_ as the Chinese population is still facing a high exposure level. The lung cancer death attributable to PM_2.5_ declined in Japan but the period risk in this country still be increasing. This is possibly related to long-term exposure to particulate matter causing health problems, such as normal lung function not being restored even after the improvement of air pollution in Japan (45). It is therefore essential for countries in East Asia to prevent air pollution.

This study has limitations. Although GBD 2019 collected missing data and improved the quality of data and its comparability by modifying and adjusting data sources and collection and evaluation methods, there might be uncertainty about exposure estimates, as there was no measurement in some areas or the data was not available in the GBD 2019 study. Therefore, the results should be carefully interpreted for these countries. Second, the majority of air quality monitoring stations were located in urban areas where air pollution mean concentrations are expected to be high. Third, the APC analysis only takes the effects of age, period, and cohort into account and does not further analyze other risk factors underlying lung cancer death.

## Conclusions

Our study showed that ambient air pollution could impose a substantial burden in terms of lung cancer death in East Asia, where China is facing the highest attributable death rate for lung cancer caused by PM_2.5_. An increasing trend of lung cancer death attributed to PM_2.5_ was observed in East Asia from 1990 to 2019, except for Japan and the Republic of Korea. However, the period effect nevertheless suggests a remarkably increasing risk in China, the Democratic People's Republic of Korea, and Japan in the long-term, and overall reduction of air pollution would have significant benefits for the health of the populations in these countries.

## Data Availability Statement

The original contributions presented in the study are included in the article/Supplementary Material, further inquiries can be directed to the corresponding author/s.

## Author Contributions

CY conceived of the study and participated in its design and coordination. XL led data collection and analysis, wrote the original draft, and oversaw editing of the final manuscript. All authors contributed to the drafting and revision of the article, read, and approved the final manuscript.

## Funding

This work was funded by the Wuhan Medical Research Program of Joint Fund of Hubei Health Committee (grant number WJ2019H304), the National Key Research and Development Program of China (grant numbers 2018YFC1315302 and 2017YFC1200502), and the National Natural Science Foundation of China (grant number 81773552).

## Conflict of Interest

The authors declare that the research was conducted in the absence of any commercial or financial relationships that could be construed as a potential conflict of interest.

## Publisher's Note

All claims expressed in this article are solely those of the authors and do not necessarily represent those of their affiliated organizations, or those of the publisher, the editors and the reviewers. Any product that may be evaluated in this article, or claim that may be made by its manufacturer, is not guaranteed or endorsed by the publisher.

## Supplementary Material

Supplemental Figure 1 National exposure in China to PM_2.5_ for 12 months during 2013-2019.

Supplemental Table 1 The age standardized rates of death and incidence lung cancer and its attributable burden to PM_2.5_ in East Asia countries compared with the global's in 2019 (%).
