# Supplementary figures and images for: Lung Cancer Death Attributable to Long-Term Ambient Particulate Matter (PM2.5) Exposure in East Asian Countries During 1990–2019
Source: Front Med (Lausanne). 2021 Oct 15;8:742076. doi: 10.3389/fmed.2021.742076 (PMC8553966; doi:10.3389/fmed.2021.742076)

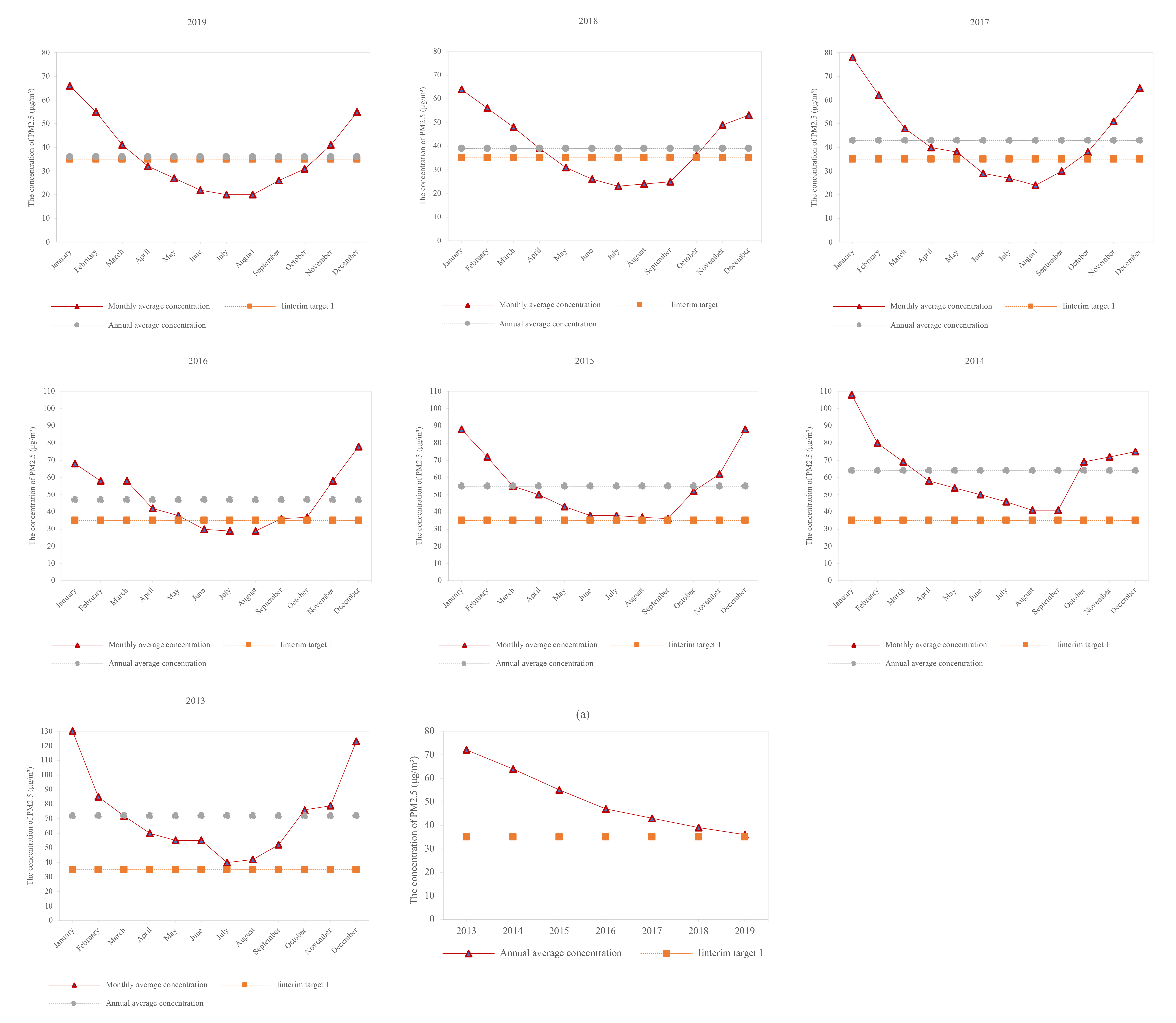

Supplement: Supplemental Figure 1 — National exposure in China to PM2.5 for 12 months during 2013-2019. [file Image_1.TIF]
