# Supplementary material for: Lung Cancer Death Attributable to Long-Term Ambient Particulate Matter (PM2.5) Exposure in East Asian Countries During 1990–2019
Source: Front Med (Lausanne). 2021 Oct 15;8:742076. doi: 10.3389/fmed.2021.742076 (PMC8553966; doi:10.3389/fmed.2021.742076)
Supplement: Supplemental Table 1 — The age standardized rates of death and incidence lung cancer and its attributable burden to PM2.5 in East Asia countries compared with the global's in 2019 (%). [file Table_1.DOCX]

| **East Asia** | **ASDR** | | |  | **ASIR** | | |  | **Attributable death to PM_2.5_** | | | | |  | **Attributable YLL to PM_2.5_** | | | | | |
| --- | --- | --- | --- | --- | --- | --- | --- | --- | --- | --- | --- | --- | --- | --- | --- | --- | --- | --- | --- | --- |
|  | **Both sexes** | **Female** | **Male** |  | **Both sexes** | **Female** | **Male** |  | **Both sexes** | | **Female** | | **Male** |  | **Both sexes** | | **Female** | | **Male** |  |
| China | 153.68 | 152.53 | 155.43 | | 150.80 | 147.06 | 152.66 | 231.66 | | 239.46 | | 231.15 | | 221.68 | | 229.92 | | 217.31 | |  |
| Democratic People's Republic of Korea | 113.33 | 109.04 | 127.04 | | 100.80 | 94.32 | 112.90 | 104.22 | | 93.15 | | 121.74 | | 117.29 | | 103.15 | | 131.94 | |  |
| Republic of Korea | 104.45 | 87.60 | 119.67 | | 128.73 | 108.43 | 146.23 | 129.19 | | 116.79 | | 143.66 | | 108.75 | | 96.88 | | 118.14 | |  |
| Japan | 84.22 | 72.82 | 92.12 | | 110.85 | 94.65 | 121.29 | 52.43 | | 49.06 | | 55.55 | | 44.79 | | 42.08 | | 46.63 | |  |
| Mongolia | 132.46 | 91.37 | 167.88 | | 112.75 | 74.45 | 143.75 | 157.31 | | 109.32 | | 197.73 | | 154.17 | | 99.49 | | 192.17 | |  |

Supplemental Table 1 The age standardized rates of death and incidence lung cancer and its attributable burden to PM_2.5_ in East Asia countries compared with the global’s in 2019(%)
